# Spatiotemporal modelling and mapping of the bubonic plague epidemic in India

**DOI:** 10.1186/1476-072X-5-12

**Published:** 2006-03-17

**Authors:** Hwa-Lung Yu, George Christakos

**Affiliations:** 1 Department of Geography, 314 Storm Hall, San Diego State University, 5500 Campanile Drive, San Diego, CA 92182-4493, USA

## Abstract

**Background:**

This work studies the spatiotemporal evolution of bubonic plague in India during 1896–1906 using stochastic concepts and geographical information science techniques. In the past, most investigations focused on selected cities to conduct different kinds of studies, such as the ecology of rats. No detailed maps existed incorporating the space-time dependence structure and uncertainty sources of the epidemic system and providing a composite space-time picture of the disease propagation characteristics.

**Results:**

Informative spatiotemporal maps were generated that represented mortality rates and geographical spread of the disease, and epidemic indicator plots were derived that offered meaningful characterizations of the spatiotemporal disease distribution. The bubonic plague in India exhibited strong seasonal and geographical features. During its entire duration, the plague continued to invade new geographical areas, while it followed a re-emergence pattern at many localities; its rate changed significantly during each year and the mortality distribution exhibited space-time heterogeneous patterns; prevalence usually occurred in the autumn and spring, whereas the plague stopped moving towards new locations during the summers.

**Conclusion:**

Modern stochastic modelling and geographical information science provide powerful means to study the spatiotemporal distribution of the bubonic plague epidemic under conditions of uncertainty and multi-sourced databases; to account for various forms of interdisciplinary knowledge; and to generate informative space-time maps of mortality rates and propagation patterns. To the best of our knowledge, this kind of plague maps and plots become available for the first time, thus providing novel perspectives concerning the distribution and space-time propagation of the deadly epidemic. Furthermore, systematic maps and indicator plots make possible the comparison of the spatial-temporal propagation patterns of different diseases.

## Background

Epidemics can have profound effects upon human populations, states and governments [1]. The bubonic plague started in southern China in 1855, but the situation was rather ignored by the rest of the world (in part because of the 1851–1864 Taiping rebellion in the area; [2]). But, when the plague reached Hong Kong and Canton in 1894, western authorities decided to intervene to avoid a worldwide epidemic [3]. Unfortunately, they were only partly successful, as the epidemic spread primarily in India with serious consequences [4,5]. It took more than 10 years to conclude that the bubonic plague was a disease transmitted by fleas. About 10% of the infected fleas develop esophagus and gizzard blockage due to the highly abnormal reproduction of bacteria after sucking blood from infected rats. The blockage makes the fleas hungry, since the blood cannot reach the intestines for digestion, which implies that the fleas start biting at a higher rate. The bubonic plague mechanism requires at least one species of rodents that is resistant to the bacteria and another one that is not. The resistant rodents keep the disease endemic by hosting the infested fleas. The infection is passed from the fleas to their victims by means of the blocked fleas that regurgitate infected blood at biting. As the sensitive rodents are infected, they die. Then the fleas, facing a shortage of their preferred host, move onto people, thus triggering the epidemic. The disease makes its appearance in humans within a week following the biting by infected fleas. The causative agent of the bubonic plague is the Yersinia pestis (Y. pestis) bacillus. Multiplication of the bacteria produces the characteristic "bubo" (swollen, painful lymph node). Bacteremia follows, causing death in about 75% of those affected.

**Figure 1:**
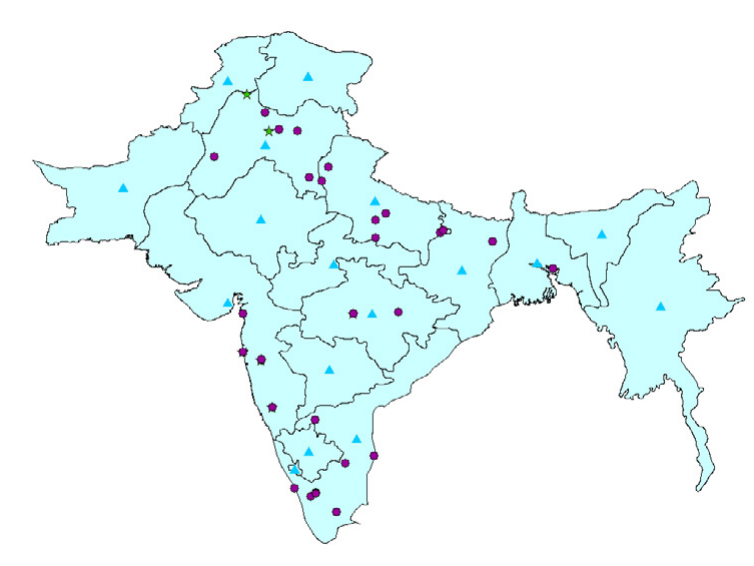
**The bubonic plague data distribution**. Circles denote yearly city data, triangles denote yearly or monthly provincial data information and stars denote monthly city data.

The Indian bubonic plague characteristics (seasonal effects, the role of rats and fleas etc.) have been well documented [6]. However, most investigations focused on selected cities to conduct different kinds of studies, such as the ecology of rats. As a result, the monthly or yearly mortality information contained in the bubonic plague database refers only to these cities; also, most of the plague data were organized at the provincial scale (the data distribution is shown in Figure 1). To our knowledge, no detailed maps exist providing a composite space-time picture of the disease propagation characteristics across space-time. In order to produce systematic space-time maps of the epidemic, it is necessary to use a rigorous methodology that makes optimal use of the provincial monthly mortality information to support a monthly database construction. At the same time, the various uncertainty sources characterizing the propagation of the epidemic must be adequately accounted by the space-time methodology.

## Results

### Spatiotemporal maps of bubonic plague mortality

**Figure 2:**
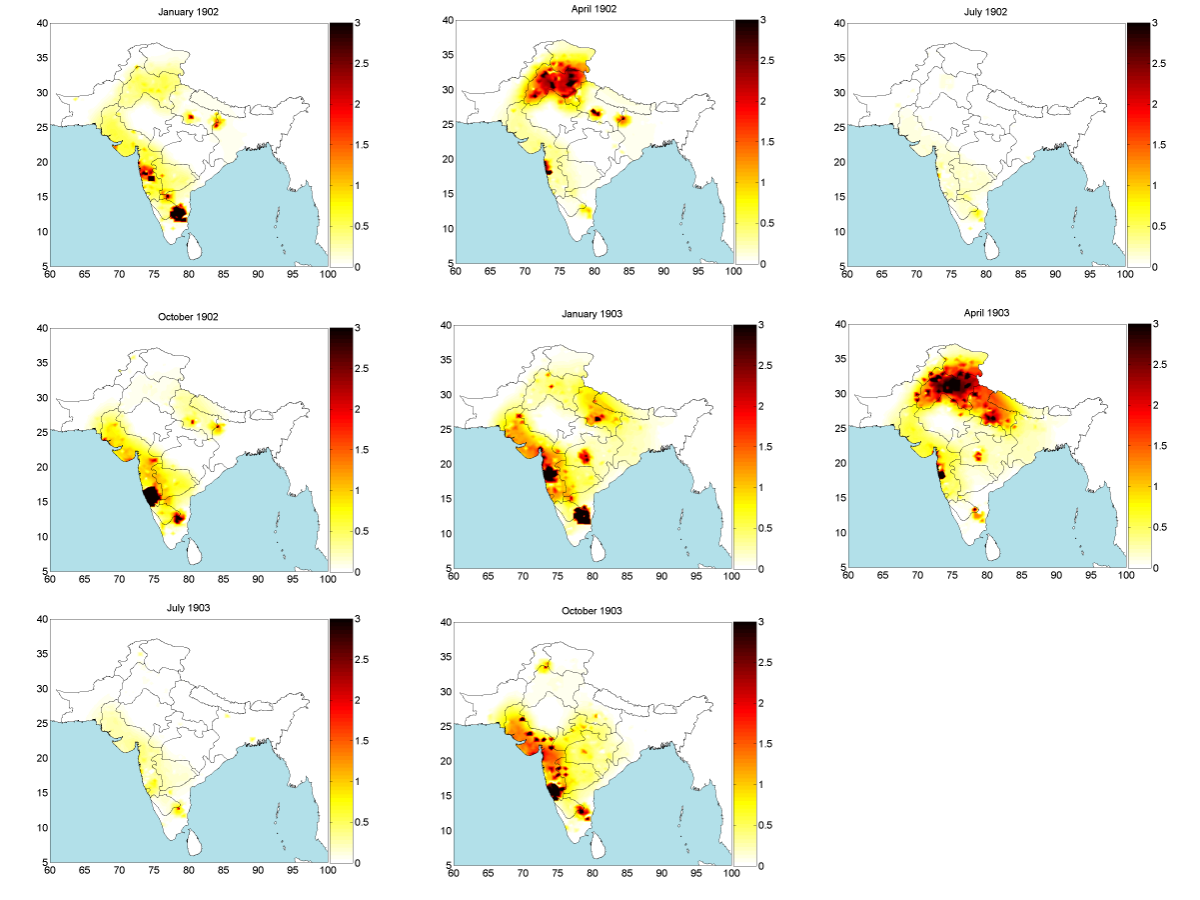
Space-time mortality rate maps during 1902–1903.

Monthly mortality rates for the period September 1896-October 1906 were collected or generated using the proposed space-time method, leading to a complete set of 122 mortality maps in space-time. The maps allow displaying and studying in detail the geographical distribution, temporal evolution and seasonal effects of the deadly epidemic. Due to space limitations, a small subset of these maps is plotted in Figure 2 (although a complete set is available). The space-time method generates the complete mortality pdf, *f*_*K*_, at each space-time point over India; from these pdf, characteristic values (mode, mean, median) can be derived. The maps in Figure 2 represent the mean values, , of the corresponding pdf.

The territory shown in these maps is India in the 1900s, excluding a part of Burma. For historic reasons, the provincial information in the database is slightly different from the information shown in the maps. E.g., the Bengal presidency was divided into 3 provinces: Bihar/Orissa, Bengal, and Assam [7]. The reason for this database division is that in 1900 these provinces were still parts of the Bengal presidency. However, in 1905 the Bengal presidency was partitioned into Bengal and Assam, whereas in 1912 Bihar and Orissa formed a new province. The monthly provincial mortality provided in [8] was in accordance with the later partition of provinces. The generated bubonic plague maps describe the varying mortality spread throughout India and during different time periods. On the basis of these maps, several observations are made below concerning the space-time distribution and propagation of the bubonic plague epidemic throughout India.

**Figure 3:**
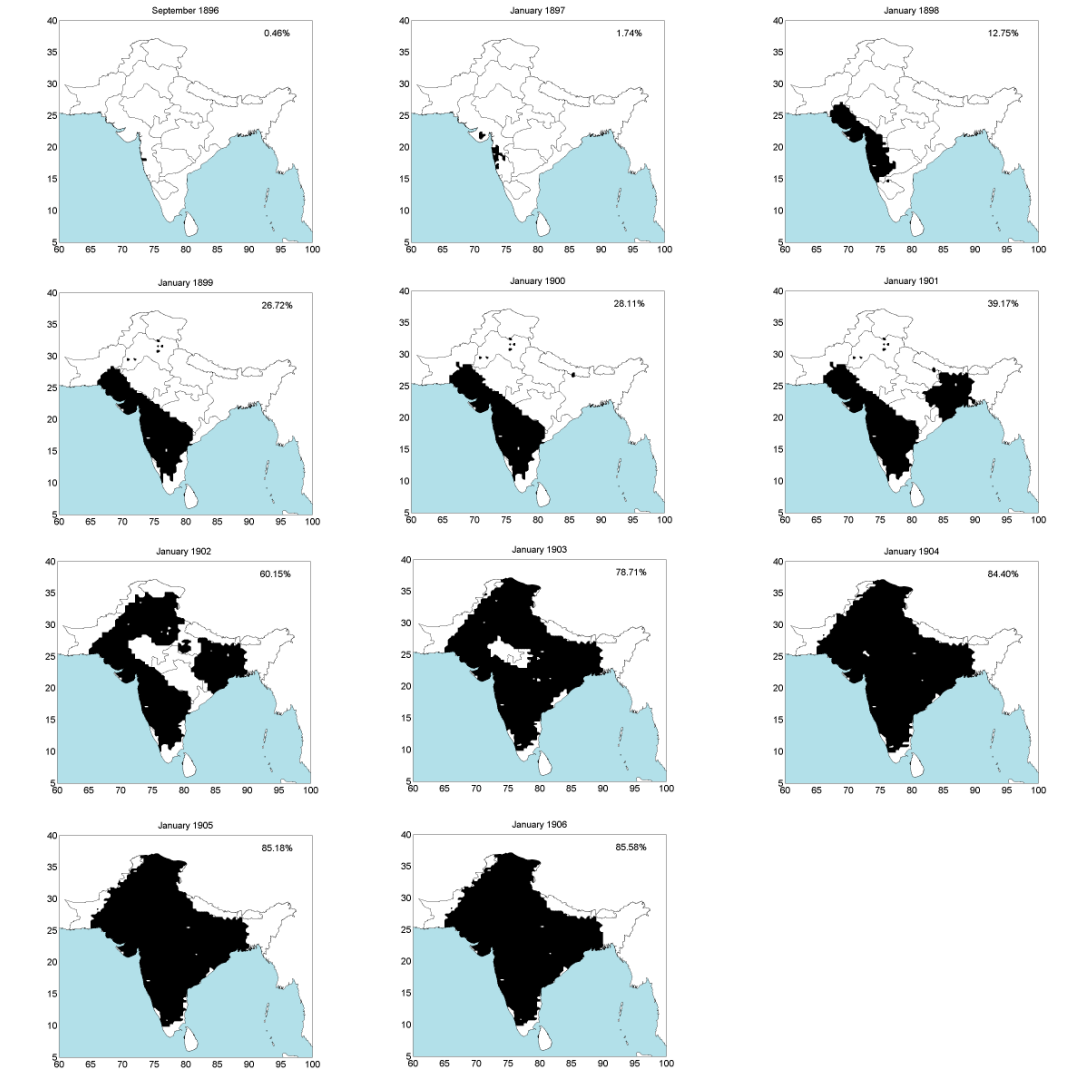
**Space-time area epidemic maps (1896–1906)**. Black area indicates the cumulated infected area. The number in top-right corner denote the percentage of entire India which was infected.

The bubonic plague started at a port: Bombay City (1896). Then, it gradually moved inland, like a cloud of varying size, depending on the geographical area (the cloud analogy aims to describe an epidemic propagation advancing but at the same time disappearing and reappearing in infected areas). Although most of India was visited by the plague (also, Figure 3 below), during the first decade of the epidemic a few regions were spared: (i) Assam (it is believed that life style and the local population conditions provided an unfavorable environment for the Rattus rattus to spread the plague) [7: 348–351]. (ii) Eastern and southern parts of the Madras presidency (the specific species of fleas limited the bubonic plague infection only among the rats) [7: 357–360]. (iii) Baluchistan in the western part of India, which today is Pakistan (because of its mountainous terrain, the temperature in Baluchistan usually falls to 0°F during the winter which prevents the fleas from breeding).

As the mortality patterns show, in its first year (1896) the plague stayed close to the Bombay City, moving only slightly along the coastal line. After the initial outbreak, the bubonic plague moved East and South into the Bombay Presidency (September 1897); the epidemic hit was severe in parts of this area and lasted until February 1898. At the same time, the northern part of the Bombay Presidency exhibited relatively lower mortality. The second epidemic wave receded in early summer 1898. In June 1898 the mortality values rose dramatically in the southern part of the Bombay Presidency and the epidemic lasted until the following spring. By September 1898 the bubonic plague had spread throughout the entire Bombay Presidency; in fact, for the first time it went past the Presidency boundaries and attacked neighboring areas, namely Hyberabad and Mysore.

In early spring 1899 the Bombay city suffered another bout of the epidemic that lasted until May. Although the epidemic showed signs of temporary recession, it started rising again in June 1899 at the southern parts of the Bombay Presidency. During the rest of the year the epidemic spread throughout the entire Bombay Presidency. The peak of the epidemic was observed in September 1899 and by December the infected area was negligible in most of the region. Nonetheless, in the western coastline and close to the Bombay city, the plague rose again and remained consistently strong starting in January 1900 and ending in May 1900, thus showing a repeating pattern of two peaks per year (unlike most other regions that experienced only one peak per year). The space-time maps indicate that the first visit of the epidemic in the Bengal province (Bihar/Orissa) occurred during March 1900. On the basis of the mortality numbers, one may deduce that the infection was relatively mild compared to that of the Bombay city area (Bombay city, Poona, Kirkee etc.). In the Bombay city the epidemic ended the month of May 1990. In July, however, the epidemic re-emerged in the southern part of the Bombay Presidency (Belgaum). Also, the epidemic spread to the North of the Bombay city in August, and the following September it moved East toward Hyderabad and Mysore. The epidemic slowed down in the Bombay Presidency during the month of December 1900.

In the beginning of 1901, the epidemic first passed the Mysore area and reached the Madras Presidency (Vaniyambadi). As the mortality maps show, the bubonic plague also recurred in the Bengal area and was more severe this time. The epidemic in the Bombay city had never completely disappeared by the end of 1900, and it made a strong reappearance in February 1901. Following the relatively calm months of May and June, high mortality was recorded in southern Bombay (August 1901), followed by the severe infection of the entire Bombay Presidency. By the end of 1901, the severity of the disease was reduced in the southern part of the Bombay Presidency, but the Bombay region displayed high mortality during December 1901. In the meantime, the epidemic re-emerged in the Madras Presidency (October 1901). During 1901 winter several other provinces in northern India had been visited by the plague (Punjab, United Provinces, Bihar/Orissa).

The space-time maps show that in early 1902 the bubonic plague became, once more, a severe epidemic in various parts of India. In Bombay city the epidemic reached its mortality peak during February 1902 and ended in June 1902. In northern India (Punjab and United Provinces) the mortality peak occurred during March-April and it also ended in June 1902. As for the southern India (inland Madras Presidency), the disease peak was observed in December 1901 and January 1902, whereas the plague receded in May 1902. Similar to the epidemic pattern of 1901-1902, the southern part of the Bombay Presidency was also the first place in which the plague recurred in August 1902, whereas peak mortality was again reached in Bombay city and the inland Madras Presidency during December 1902. The plague then moved to the North occupying the entire Bombay, Punjab, and United Provinces. In December 1902 the first infection took place in the Central Provinces. Interestingly, the mortality distribution in Bombay city demonstrated a similar behavior, which consisted of an increasing trend during the winter that reached a peak in the following spring and then ended in late spring of the same year.

The mortality maps reveal that in southern India the plague lasted until May 1903 and in northern India until June 1903. Following the last epidemic in Punjab, a related pattern was repeated in 1903. The mortality peak of bubonic plague occurred in the southern Bombay Presidency during September of the same year. In November 1903 the epidemic spread toward the inland Madras Presidency and the northern part of Bombay Presidency (Surat). Following this trend, the plague subsequently moved into the Central Provinces and the Bombay city (December 1903-January 1904), and then went further up north to the United Provinces and Bengal area (March 1904) and Punjab (April 1904).

In early spring 1904, the bubonic plague had infected almost the entire northern India (Punjab, Rajputana, United Provinces, Bengal and Kashimir). Punjab was the most severely hit province and the one where the epidemic lasted the longest. In contrast, Rajputana suffered a relatively milder blow from the plague. From a topographical viewpoint, the Thar desert, the Mount Abu, the Aravalli range, and the tropical forests in this region are not propitious to fleas or rats commuting. However, Punjab is an extensively flat plain, open to the east and south, and its climate and temperature are favorable for flea breeding. In south India (Bombay Presidency, Mysore and Hyderabad) the plague re-appeared in July 1904 and then started moving north. Following the pattern of the previous years, the epidemic peaked at the southern Bombay Presidency during September; then, in late 1904 it went north into Bombay city, the Surat region, Central India and the Central Provinces; during March-April 1904 the epidemic moved to Punjab, United Provinces and Bengal reaching its mortality peak. A difference with the previous years was that, this time, the epidemic did not infect the Madras Presidency.

On the basis of the mortality maps of the years 1904–1905, one observes that the epidemic spread across India from south to north. High mortality rates were recorded at the Bombay Presidency, Punjab, United Provinces and Bengal. Despite this trend, infection rates were relatively modest in Mysore, Hyderabad, Central India, Rajputana and the Central Provinces (except in the Nagpur city). The topography of India may explain the close correlation between devastation degree and the terrain variability. The hills and mountains of central India are a rather unfavorable migration environment for fleas and rats compared to the flat plains of the Bombay Presidency, Punjab, the United Provinces and the Bengal Presidency. In the fall of 1905, the Bombay Presidency had a comparatively mild epidemic. After that, the disease moved into northern India.

In January 1906 the epidemic relocated to the Central Provinces. During March-April the plague moved again into the northern India where it lasted until June. As it had happened in previous years, the Bombay city suffered another epidemic attack during the spring of 1906. Noticeably, during the epidemic years the Nagpur city displayed a different behavior than the rest of the region. Nagpur city was the capital of the Central Provinces and the trade centre of middle India; it also was one of the few cities connected with the Great India Peninsula Railway (GIP) system, [9], at the time. The convenience provided by the developed transportation means may have been an influential factor in the space-time spread of bubonic plague. The railway map of India in 1893 offers some hints concerning the close association between the railway system and the epidemic distribution. In the early 1900s, India was divided into British India (Bombay Presidency, Punjab, United Provinces, Bengal Presidency etc.), as well as other native India states (Hyderabad, Mysore, Central India etc.). GIP offered good connections for most of the British India region; however, the native India parts were more difficult to reach than the British India region. Therefore, the transportation infrastructure may provide some insight on why British India generally suffered more by bubonic plague than the native India. The railway also increased the spreading speed of the bubonic plague during 1902–1903, as is noticed in the space-time mortality maps of these years. Cawnpore (a railway junction in the United Provinces) and Bhagalpur (the administrative and marketing center in Bihar with railroad connection) were both infected by more severe bubonic plague attacks than the remaining areas in the region.

### Spatiotemporal maps of geographical evolution

The selection of maps in Figure 3 shows the geographical evolution of the epidemic during different times (a complete set of more than 120 maps is available but is not plotted here, due to space limitations). The maps are derived on the basis of the mortality distributions available during the construction of the spatiotemporal mortality maps. Let *P*[*M*_*p *_> 0.01%] denote the probability that mortality *M *at a given space-time point ***p ***exceeds the 0.01% threshold. A location is considered infected when *P*[*M*_*p *_> 0.009%]>0.65 at that location. Several alternative sets of maps can be constructed by choosing different mortality and probability thresholds. This flexibility of geographical information science ([10]) allows one to focus on important space-time features of the bubonic plague epidemic.

According to the geographical evolution maps the dispersion of the bubonic plague in India would either slow down or come to a complete halt from April to August in each epidemic year. Another feature of the disease evolution made apparent from the mortality maps is that the epidemic tended to stop or slow down during certain periods of the year, even if at the time the epidemic would tend to restart or would still be going through a severe stage in a region.

### Spatiotemporal epidemic pattern

**Figure 4:**
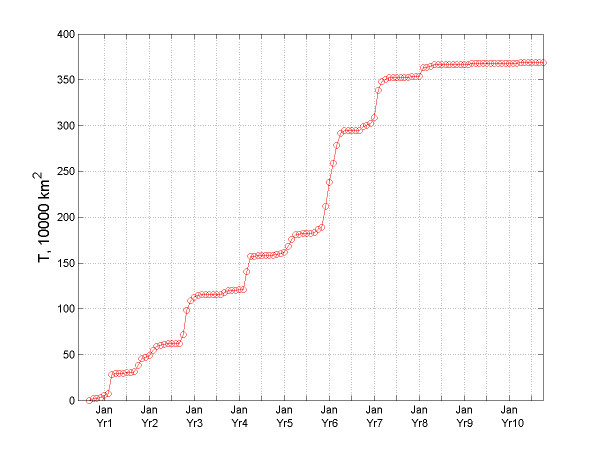
Temporal variation of the T-indicator for bubonic plague.

The temporal variation of the total infected area indicator, *T*(*t*), for the Indian epidemic is shown in Figure 4. During its entire duration, the bubonic plague invaded new geographical areas at an average rate of about 4 × 10^4 ^km^2^/month. Considered in the context of local time intervals, the *T*-curve of the bubonic plague spread consists of two parts (Figure 4): The slope of the one part is close to zero, which signifies a halt in the plague spread; the second part, however, exhibits a steep slope indicating that the bubonic plague would spread very fast at a time of high activity. In fact, the propagation rate during the months of December 1901-May 1902 is the fastest observed for the epidemic during any time period.

**Figure 5:**
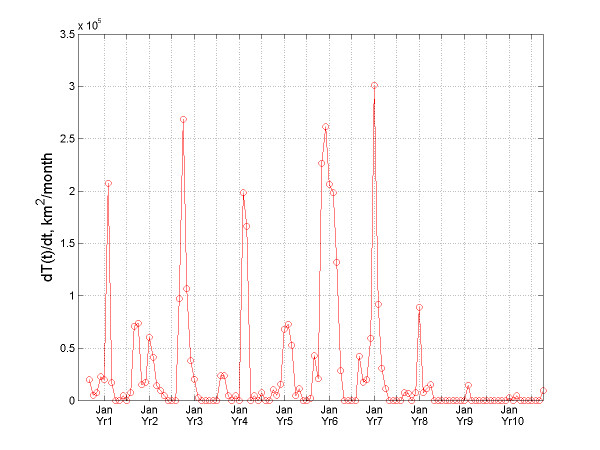
Temporal variation of the dT/dt-indicator for bubonic plague.

**Figure 6:**
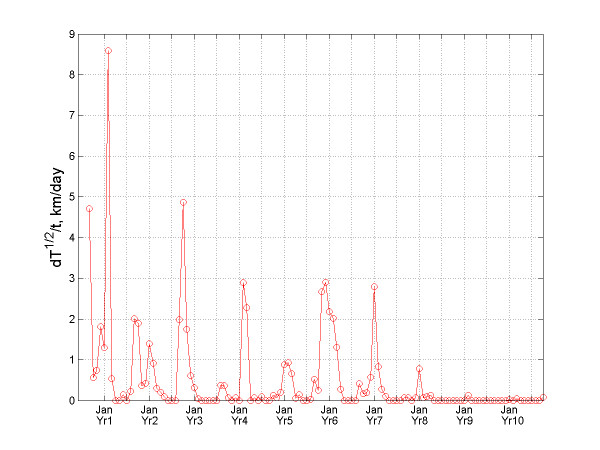
Temporal variation of the dT^1/2^/dt-indicator for bubonic plague.

An interesting phenomenon is observed in Figure 5 that plots the indicator *T*(*t*) vs. time. This quantity can be thought of as the "instant speed" of the total infection area expansion. The rate for the bubonic plague changed significantly within each year. The variation of the "instant rate" for the plague epidemic indicates that it exhibited a seasonal effect. The five most aggressive spread rates of bubonic plague occurred in March 1897, November 1898, March 1900, January 1902, and February 1903; whereas the four less aggressive rates occurred in November 1897, February 1898, March 1901, and February 1904. Before each large outburst, it took 1–2 years for bubonic plague to preserve its energy, and for that time it would stay static at the same locations. The huge expansion bursts of the total infected area *T*(*t*) took place mainly during the spring. Similar observations can be made about Figure 6, which plots *T*(*t*)^1/2 ^vs. time providing a measure of the speed of the total infected area *T*(*t*).

**Figure 7:**
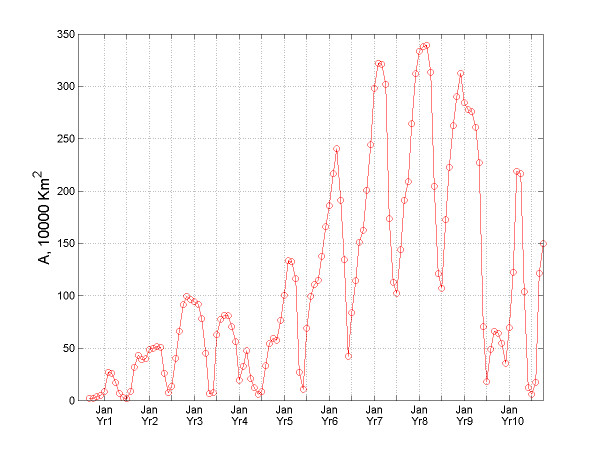
Temporal variation of the A-indicator for bubonic plague.

**Figure 8:**
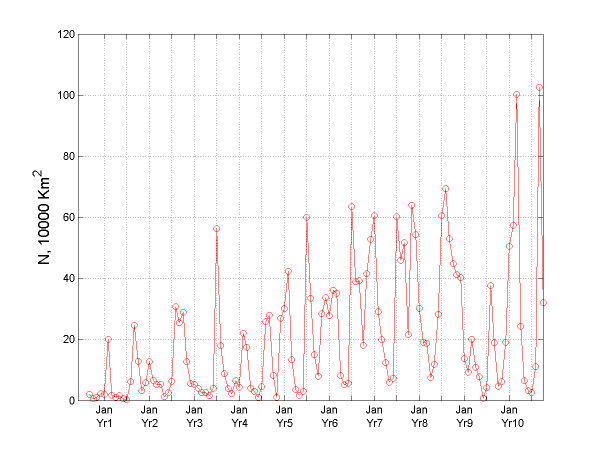
Temporal variation of the N-indicator for bubonic plague.

**Figure 9:**
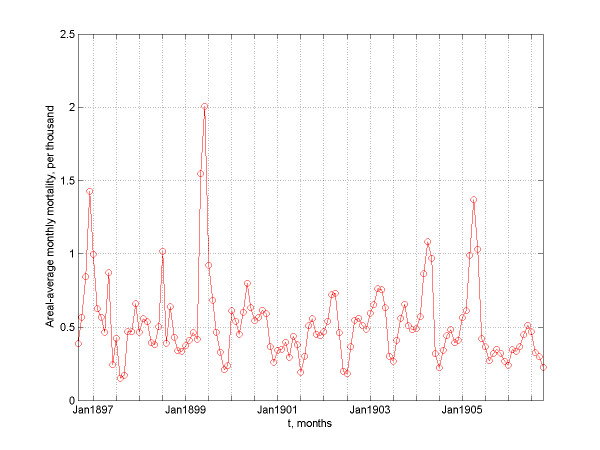
The areal-averaged monthly mortality rates of bubonic plague vs time.

According to the geographic evolution maps of the epidemic, it is evident that the bubonic plague ravaged across almost its entire space – the subcontinent of India. From the plot of *A*(*t*) vs. *t *(Figure 7) it is clear that bubonic plague grew larger and larger during the years, and at certain times it occupied most regions of India. The reason for this is that the plague followed a re-emergence pattern from its prior infection in the geographical locations. The plot of *N*(*t*) vs. *t *in Figure 8 gives some insight about the relocation mechanism for the bubonic plague epidemic. The ragged bubonic plague curve shows that the plague would change its location twice a year, i.e., almost every spring and fall. The area-averaged monthly mortality variation plot for bubonic plague is shown in Figure 9. The plot is in agreement with the results of [11], which had concluded that the plague mortality during the years 1900–1905 did not exceed 0.4% per year.

Moreover, the epidemic indicators allow some interesting comparisons of the Indian epidemic vs. the medieval Black Death epidemic in Europe (for a detailed discussion of the spatiotemporal characteristics of Black Death, see [10]). E.g., during the bubonic plague's first 40 months there were only 3 times when the mortality rate was raised to levels comparable to those of the medieval Black Death; during the rest of the time, the plague either remained static geographically or it completely disappeared. As was discussed in previous sections, bubonic plague was stale in the summer and active in the spring and fall, whereas the geographical Black Death evolution tended to be slower during winter. Bubonic plague fatalities in India were minimal to none during the dry and hot months of April-August [7]. On the contrary, the Black Death casualties merely slowed down during the cold winter months; moreover, after the first summer, Black Death reached a global maximum that was followed by maxima of decreasing levels, whereas bubonic plague kept reaching new heights for several years.

## Discussion – conclusion

We introduced rigorous stochastic modelling in the spatiotemporal analysis of the bubonic plague epidemic in India during the years 1896–1906. We discussed geographical information science concepts and techniques underlying the study of the bubonic plague patterns of growth.

A variety of informative space-time maps of mortality rates and geographical disease evolution were generated that accounted for multi-sourced databases and uncertainty conditions. A series of epidemic indicators were plotted that offered meaningful characterizations of the spatiotemporal disease distribution.

The bubonic plague in India exhibited strong seasonal and geographical features. During its entire duration, the plague continued to invade new geographical areas, while it followed a re-emergence pattern at many localities; its rate changed significantly during each year and the mortality distribution exhibited space-time heterogeneous patterns; prevalence usually occurred in the autumn and spring, whereas the plague stopped moving towards new locations during the summers.

Among other things, the epidemic maps and indicators make possible the comparison of the spatiotemporal characteristics of different diseases. Such comparisons could detect discrepancies in the spatiotemporal patterns of the epidemics; it is then left to specialized investigations to seek substantive and biologically plausible explanations of these discrepancies.

## Methods

### Stochastic spatiotemporal modelling

Methodologically, the bubonic plague distribution across space-time is conceptualized as a stochastic epidemic system and the bubonic mortality rates are represented as spatiotemporal random fields (S/TRF). The geographical information technology is subsequently implemented to map these rates in the composite space-time domain of the epidemic. This is a synthetic methodology ([10]) that provides the means to understand the nature of space-time disease processes among populations of individuals.

Our understanding of past epidemics is uncertain because the information available is inconclusive and to some degree inaccurate (on occasion the available evidence appears contradictory). Therefore, in modern epidemic studies the S/TRF theory is the main tool of stochastic modelling. The S/TRF theory adequately contextualizes and evaluates the uncertainty sources concerning the space-time distribution of an epidemic. The values of a disease distribution are attributed to spatiotemporal points under conditions of uncertainty, and multiple realizations are considered that account for composite space-time disease variations together with the associated probability models. A mathematical presentation of the S/TRF theory can be found in [12,13]. Before proceeding with our discussion of the theory, let us note that mortality distribution was selected as the space-time variable characterizing the bubonic plague epidemic in India, since the available information is related (directly or indirectly) to mortality. In particular, based on data availability, the monthly mortality rate is defined as *M*_*monthly *_= *D*_*monthly*_/*TP *= *RD*_*monthly *_, where *D*_*monthly*_, *D*_*yearly *_are the numbers of plague deaths per month and per year, respectively, *TP *is the total population and *R *is the ratio of yearly plague deaths over the total population, and can be represented as *R *= *D*_*yearly*_/*TP*.

Let ***p ***= (***s***, *t*) be a point in the space-time domain; ***s ***denotes the spatial location and ***t ***the time instant under consideration (months, years etc.). In the S/TRF context one views space and time in a composite way: as a single block of space-time. In this sense one may distinguish between the separable space-time domain considered in most statistical analyses vs. the composite space-time domain of S/TRF analysis. Basically, an S/TRF *M*_*p *_= *M*_*s*,*t *_representing mortality distribution is a collection of realizations (possibilities, potentialities) for the disease values in space-time. The multiplicity of realizations allows S/TRF to account for uncertainty sources and adequately represent the spatiotemporal variation of bubonic mortality in India. Stochastically, the S/TRF model is fully characterized by its probability density function (pdf), *f*_*KB*_, generally defined as



where the subscript KB denotes the knowledge base utilized to construct the pdf (the pdf depends explicitly on the points ***p***_1_, ***p***_2_, ..., but we do not explicitly show this for convenience). Stochastic modelling of disease distributions with complicated space-time patterns entails determining the S/TRF from a single realization. Trend-free (spatially homogeneous and temporally stationary) S/TRF have been assumed widely in health geographics, because they are efficient for explicit and numerical calculations. However, the homogeneous-stationary model is not the best option for phenomena with large and complicated space-time variabilities.

In light of these issues, a class of heterogeneous S/TRF has been proposed by [12] that is considerably more general than the restricted class of homogeneous-stationary S/TRF. This new class is capable of handling complicated space-time variabilities of any size based on the following intuitive idea: The variability of an S/TRF can be characterized by means of its degree of departure from homogeneity and stationarity. This departure can be determined by a mathematical operation, in the following sense. Let *Q*_*ν*/*μ *_be a space-time operator that transforms the S/TRF *M*_*p *_into a homogeneous-stationary field *Y*_*p *_by annihilating heterogeneities of degrees *ν *in space and *μ *in time, i.e.,

*Q*_*ν*/*μ *_[*M*_*p*_] = *Y*_*p *_    (2)

In this case, the mortality field *M*_*P *_is said to be an S/TRF with spatial and temporal heterogeneity orders *ν *and *μ*, respectively (S/TRF-*ν /μ *; [12,14]). The S/TRF-*ν /μ *offers a general theoretical model of the mortality distribution that expresses the way causal influence is propagated in space-time and gives information about the bubonic plague dynamics at the scale of interest. For epidemic systems that evolve within domains containing complicated boundaries and trends, the departure of the random field from homogeneity-stationarity is expected to vary geographically. It is, thus, meaningful to construct local *Q*_*ν*/*μ *_-operators that generate homogeneous-stationary random fields *Y*_*p *_within local neighbourhoods Λ, instead of seeking global representations.

In practice, various choices of *Q*_*ν*/*μ *_-operators are possible, thus allowing considerable flexibility in the definition of the S/TRF-*ν*/*μ*. Indeed, the spatiotemporal trend of the mortality distribution *M*_*p *_inside each local neighborhood Λ of the epidemic system may involve various functions with space-time coordinates (polynomial, exponential, trigonometric etc. functions). For the purposes of the current bubonic plague study, an efficient *Q*_*ν*/*μ *_-operator is one that annihilates local heterogeneities involving composite space-time polynomials, *P*_*ν*/*μ*_, of degrees *ν *in space and *μ *in time. The parameters *ν *and *μ *provide a quantitative assessment of the rate of change of the functions modelling mortality patterns. E.g., the smaller the *P*_*ν*/*μ*_, degrees, the smaller the values of *ν *and *μ *The *ν*, *μ *also offer information about the stochastic model underlying the epidemic system. E.g., these parameters may determine how "far away" in space and "deep" in time the operator searches for information about the mortality field.

Mortality correlations across space-time are characterized by the ordinary spatiotemporal covariance *c*_*M*_(***p***, ***p'***) between any pair of points ***p ***and ***p'***. This covariance is nonhomogeneous in space and nonstationary in time, and according to the S/TRF- *ν*/*μ*, theory it can be decomposed as

*c*_*M*_(***p***, ***p'***) = *κ*_*M*_*(****p**** - ****p'****)+ P*_*ν*/*μ*_,     (3)

where *κ*_*M *_is called the generalized spatiotemporal covariance, and ***p ***- ***p' ***= (***s ***- ***s'***, *t *- *t'*). An important feature of this mortality field model is that only the *κ*_*M *_is required in spatiotemporal mapping. The *κ*_*M *_can be expressed in terms of the residual ordinary covariance *c*_*Y*_, so that given the form of *c*_*Y*_, the corresponding *κ*_*M *_is derived. Hence, the class of generalized space-time covariances is richer than that of ordinary ones.

### Knowledge bases

In epidemic studies one often needs to integrate multiple sources of information, depending on the situation. In this work, the following knowledge bases (KB) are considered: the general KB,  (core knowledge about the disease), the specificatory KB,  (site-specific hard and soft, instrument- and/or survey-based data), and the integration KB,  (the union of the - and -KB).

**Figure 10:**
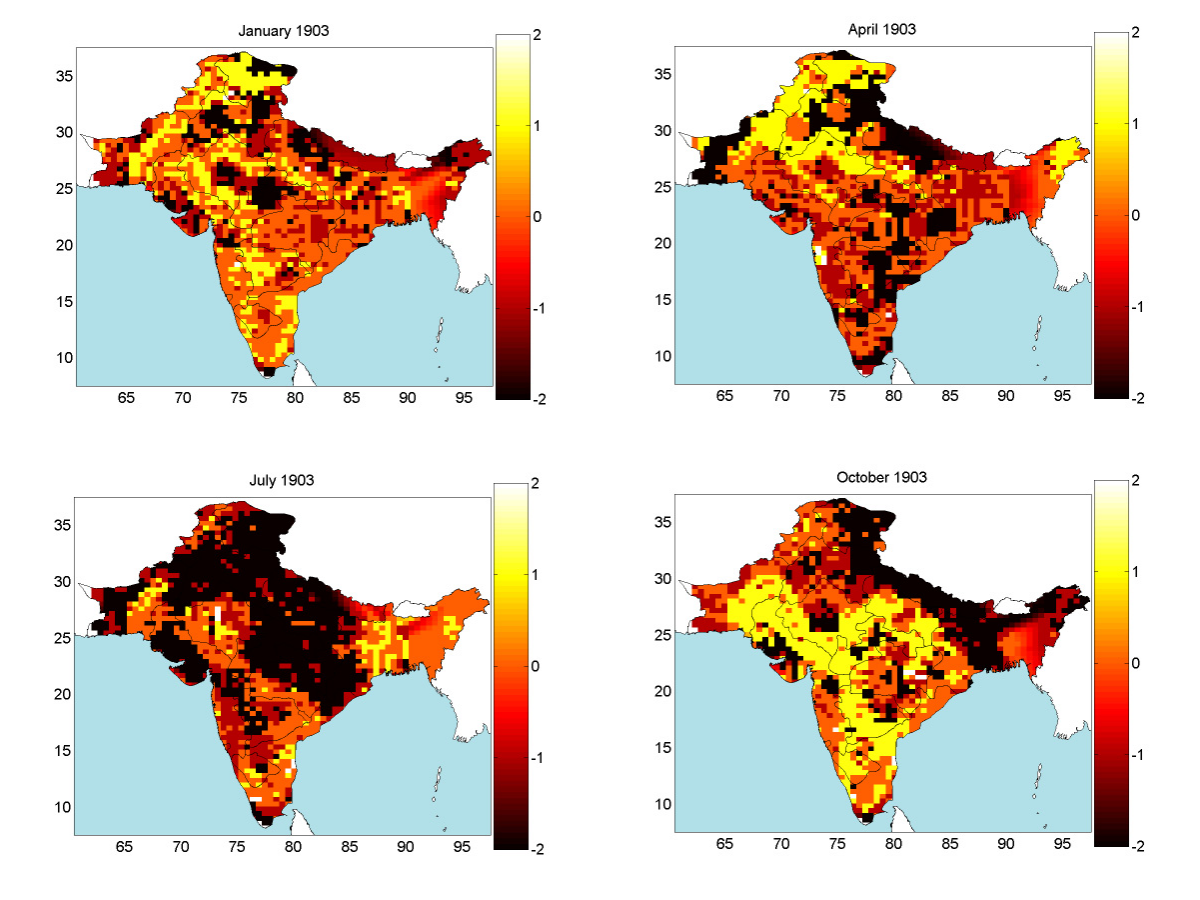
Space-time maps of the *ν*/*μ *difference in 1903.

In principle, the -KB accounts for existing scientific knowledge (theoretical models, epidemic laws etc.), but also other kinds of useful knowledge that are relevant to the epidemic system under consideration. In the present study, the scientific part of the -KB includes the S/TRF-*ν*/*μ*, theoretical model representing dependence of bubonic plague mortality across space and time (in terms of the heterogeneity orders and generalized covariance). Region-wide S/TRF parameters *ν*, and *μ*, offer insight into space-time epidemic variations across India. The calculation of the parameters in practice is made in terms of a computational procedure discussed in detail in previous publications (e.g., [13,15]). Maps showing the regionalized *ν *- *μ*, distribution over India (e.g., during the year 1903) are shown in Figure 10, thus providing information about the relative trends of mortality in space-time (*ν *- *μ *>0 implies higher degree spatial trends; *ν *- *μ *<0 implies higher degree temporal trends). Not only the spatial order *ν *changes in space, but the temporal order *μ *varies in space, as well. Spatial and temporal trends are interrelated, since the geographical propagation of the plague is also affected by temporal mechanisms. Theoretical *κ*_*X *_models assumed to express the space-time dependence of bubonic plague mortality are shown in Eq. (4) of Table 1. The coefficients *c*, *a*_*ζ*_, *b*_*ρ *_and *a*_*ρζ*_, of the models (4) must satisfy the permissibility conditions of Table 2. The values of these coefficients vary, depending on the local space-time neighborhood Λ of the epidemic system considered.

**Table 1:** Generaized covariance, *κ*_*X*_, models.

**Table 2:** Permissibility conditions for the *κ*_*X *_models of Eq. (4)

(*ν*, *μ*)	*c*	*a*_ζ_	*b*_*ρ*_	*a*_*ρ*ζ_
(0,0)	*c *≥ 0	*a*_0 _≥ 0	*b*_0 _≥ 0	*a*_00 _≥ 0
(0,1)	*c *≥ 0	*a*_0_,*a*_1 _≥ 0	*b*_0 _≥ 0	*a*_00_,*a*_01 _≥ 0
(0,2)	*c *≥ 0	*a*_0_,*a*_2 _≥ 0 *a*_1 _≥ -	*b*_0 _≥ 0	*a*_00_,*a*_01 _≥ 0 *a*_01 _≥ 0-
(1,0)	*c *≥ 0	*a*_0 _≥ 0	*b*_0_,*b*_1 _≥ 0	*a*_00_,*a*_10 _≥ 0
(1,1)	*c *≥ 0	*a*_0_,*a*_1 _≥ 0	*b*_0_,*b*_1 _≥ 0	*a*_00_,*a*_01_,*a*_10_,*a*_11 _≥ 0
(1,2)	*c *≥ 0	*a*_0_,*a*_2 _≥ 0 *a*_1 _≥ -	*b*_0_,*b*_1 _≥ 0	*a*_00_,*a*_10_,*a*_02_,*a*_12 _≥ 0 *a*_01 _≥ - *a*_11 _≥ -
(2,0)	*c *≥ 0	*a*_0 _≥ 0	*b*_0_,*b*_2 _≥ 0 *b*_1 _≥ -	*a*_00_,*a*_20 _≥ 0 *a*_10 _≥ -
(2,1)	*c *≥ 0	*a*_0_,*a*_1 _≥ 0	*b*_0_,*b*_2 _≥ 0 *b*_1 _≥ -	*a*_00_,*a*_01_,*a*_20_,*a*_21 _≥ 0 *a*_10 _≥ - *a*_11 _≥ -
(2,2)	*c *≥ 0	*a*_0_,*a*_2 _≥ 0 *a*_1 _≥ -	*b*_0_,*b*_2 _≥ 0, *b*_1 _≥ -	*a*_00_,*a*_02_,*a*_20_,*a*_22 _≥ 0 *a*_10 _≥ - *a*_01 _≥ - *a*_11 _≥ - *a*_21 _≥ - *a*_12 _≥ -

The -KB includes various information sources relevant to the Indian bubonic plague, starting the year 1896 [6,8,16]. The following terminology is used: *P*_*s*,*t *_= population; ,  = yearly and monthly city mortalities rates; ,  = yearly and monthly provincial mortality rates; ,  = number of city deaths per year and per month; ,  = number of provincial deaths per year and per month. Also, the following parameters are introduced: ; , ; and , ; with  = average numbers of city and provincial deaths per month. In light of the above considerations, the  -KB can be effectively divided into several sub-groups, as discussed in detail in the Appendix. The estimation of the  values at each geographical location and time instant involves certain uncertainty sources:

*i*. Uncertainty in the calculation of the  ratios. This is due to: (1)  values at a given year must be calculated from  data, and (2)  values are approximated from the available provincial data at different times.

*ii*. Uncertainty due to the change of spatial support (province vs. city). This situation occurs when: (1) the  values are calculated from the  data, or (2) the  values are calculated from the  data.

*iii*. Uncertainty due to the approximate readings of the charts. This is the case when one seeks to interpolate between the chart values etc.

An elementary formula of monthly city mortality is . Due to the incompleteness of the ,  and  datasets, one should account for the relevant uncertainties as described in Table 3 (the Gaussian law is used to define different uncertainty sources; , and  = mean monthly ratio during the 1899–1906 at a given province). Variances are due mainly to change of support: the  (respectively, ) expresses the uncertainty due to extrapolation from monthly provincial to monthly city ratios (respectively, mortality) at a given year (respectively, during 1899–1906);  is due to differences between the available yearly mortality city data and the mortality values approximated by means of the charts; and  is the uncertainty due to differences between monthly mortality city values and the mean monthly mortality provincial values.

**Table 3:** Uncertainty sources and assessment of the various bubonic plague datasets.

Dataset – Appendix B	Uncertainty Sources			
*a*	*i*(1)	Known		N/A
*b*	*ii*(1)	N/A	Not available	
*c*	*iii*	Known		N/A
*d*	*ii*(2), *iii*			N/A
*e*	N/A	N/A	N/A	
*f*	*i*(2), *ii*(2)			N/A
*g*	*i*(2)	Known		N/A

N/A = Not available

For mathematical formalization purposes, the vector ***m***_*map *_= (***m***_*hard*_, ***m***_*soft*_, ***m***_*k*_) denotes a possible mortality field realization associated with the space-time point vector ***p***_*map *_= (***p***_*hard*_, ***p***_*soft*_, ***p***_*k*_) within each neighborhood Λ of the epidemic domain. The ***m***_*map *_includes hard data (exact observations) ***m***_*hard *_= (*m*_1_, ..., ) at points ***p***_*hard *_= (*p*_1_, ..., ), soft data (different kinds of uncertain observations) ***m***_*soft *_= (, ..., *m*_*d*_) at ***p***_*soft *_= (, ..., ***p***_*d*_), and the estimated mortality value *χ*_*k *_at the point ***p***_*k*_. It is certainly possible to consider several mapping points in a neighborhood, in which case one is dealing with the vectors *χ*_*k *_= () and ***p***_*k *_= ().

### The BME- *ν*/*μ *technique

The geographical information technology involves a variety of advanced space-time mapping techniques (for details, see [17]). In this study, the BME- *ν*/*μ*, technique (Bayesian Maximum Entropy of order *ν*/*μ*,) has been employed, which has a number of distinguished features including the following [15,18]:

- it accounts for the uncertainty features of the epidemic system in a composite space-time domain (unlike, e.g., spatial prediction techniques that focus on purely spatial domains);

- it imposes no restriction on the shape of the probability laws and the predictor forms (non-Gaussian laws and non-linear predictors are automatically incorporated);

- it assimilates a variety of core knowledge bases and site specific, multi-sourced datasets; and

- it derives several previous techniques of epidemic modelling (statistical regression, spatial prediction, kriging etc.) as its limited cases.

In light of these important features, the mapping of the mortality distribution can be more informative and realistic than the maps generated by conventional methods that do not possess the theoretical and applied qualities of BME-*ν*/*μ*, (most of the conventional methods can use limited datasets, the linearity and normality restrictions apply etc.).

Within each neighborhood Λ of the geographical domain of the epidemic, the BME-*ν*/*μ*, mapping of the bubonic plague follows three knowledge synthesis stages:

***a*. **At the so-called structural stage, a pdf  is constructed on the basis of the -KB available, i.e.,



where, *Q *= *Q*(***m***_*map*_) is the *ν*/*μ*, -operator, *z *is the degree of freedom determined by the spatiotemporal trend, and *κ*_*map *_is the generalized covariance matrix between the points ***p***_*map*_.

***b*. **At the specificatory stage, the  -KB considers hard and/or soft data (in the form of intervals and local probability distributions). Operationally, transformations of the following type are considered , where  and *D *denote, respectively, a specificatory operator and information range determined by  -KB. For illustration, Table 4 presents some examples of -KB together with the , *D *associated with the displayed soft data;  is the cumulative distribution function derived from  at the soft data points, ***I ***is a vector of mortality intervals at these points, and ***I***_*k *_is a vector of interval values at the estimation points themselves.

**Table 4:** Examples of soft data with integration domain *D *and operator .

	***D***	
Interval	***I***	***m***_*soft*_
Probabilistic	***I***	(***m***_*soft*_)
Functional	***I ***∪ ***I***_*k*_	(***m***_*soft*_,***m***_*k*_)

***c*. **At the integration stage, the - and  -KB are combined to yield the integrated pdf  at each mapping point ***p***_*k *_using the operational Bayes formula



where *A *is a normalization constant. From the integration pdf (6), various kinds of bubonic mortality estimates can be readily derived across space-time. E.g., the mean estimate, ; and the mode estimate, . The uncertainty of the mortality estimates may be assessed in terms of the mean squared estimation error, *σ*_*k*_, at each space-time mapping point.

## Competing interests

The author(s) declare that they have no competing interests.

## Authors' contributions

Both of authors were involved in the data analysis and processing, manuscript writing and editing.

## Appendix 1

**The Database of Bubonic Plague in India**. The -KB of the bubonic plague epidemic in India is effectively divided into seven sub-groups:

(*a*) Dataset consisting of  and ratio  values derived on the basis of the information available in [8]. The  data were decomposed into  values by means of  = . For cities in the Punjab region, the  values were calculated directly from the number of plague deaths  and the provincial population *P*_***s***,*t *_available in [6] (1912: 105).

(*b*) Dataset including  values ([8]). Monthly mortality is defined as  = . The  data are available starting July 1898; thus,  ratios are available after 1899.

(*c*) Dataset consisting of  values ([6], 1912: 258) and the chart of the  ratios ([6], 1908: 301). Plague mortality is expressed in the charts in terms of percentages relative to the mean plague deaths during the period of interest. Mean monthly mortalities are calculated from the given yearly mortalities, which then lead to the  values. The dataset includes the city of Bombay, which in 1896 was the starting point of the plague outbreak in India. The yearly mortality data and ratio charts in [6] (1912: 258 and 301) do not include the year 1896, although the DSAL (Digital South Asian Library) gives geographical  values for this year. The  values provided in [19] (1900: 11) do not always agree with information obtained from other sources. Thus, data generated from this source were used to calculate the  ratios and subsequently to obtain the  values from the  data.

(*d*) Dataset consisting of  charts. The charts show the variation of city plague mortality but, as was mentioned above, they only provide values relative to the mean monthly death rate. Provincial yearly mean mortality, , served as the mean monthly city mortality, , where *N *= number of years considered. Monthly city mortality was calculated by .

(*e*) A dataset consisting of  values, which can be treated as hard data (since it is directly available). To transfer these values in to mortality data, the population information was calculated from the total plague deaths per year and the yearly mortality provided in [6] (1912: 258).

(*f*) Dataset of  values obtained from DSAL, which provides the population and number of plague deaths during each year. Using the mean monthly mortality at each province for 1899–1906, the yearly mortality was transferred into the required , where  for each month.

(*g*) Dataset consisting of  values. The city data before the year 1899 were decomposed into monthly values as follows, .
